# A comparative analysis of mycotoxin contamination of supermarket and premium brand pelleted dog food in Durban, South Africa

**DOI:** 10.4102/jsava.v88i0.1488

**Published:** 2017-10-06

**Authors:** Sanil D. Singh, Anil A. Chuturgoon

**Affiliations:** 1 Biomedical Research Unit, University of KwaZulu-Natal, South Africa; 2 Discipline of Medical Biochemistry, University of KwaZulu-Natal, South Africa

## Abstract

Dry pelleted dog food in the South African market is available via supermarkets, pet stores (standard brands [SBs]) and veterinary channels (premium brands [PBs]). For the purpose of this study, the supermarket channel included the cheaper quality foods and PBs were sold via the veterinary channel (*n* = 20). These feeds were analysed for four main mycotoxins (aflatoxins [AF], fumonisin [FB], ochratoxin A [OTA] and zearalenone [ZEA]) using standard well-described extraction, characterisation and quantitation processes. Irrespective of the brand or marketing channel, all foods were contaminated with fungi (mainly *Aspergillus flavus, Aspergillus fumigatus* and *Aspergillus parasiticus*) and mycotoxins (most prevalent being aflatoxins and fumonisins). This was observed in all 20 samples irrespective of the marketing channel or perceived quality. Also, many samples within each marketing channel failed the 10 ppb limit for aflatoxin set by regulations in South Africa. Although fumonisin was detected in all samples, a single sample failed the Food and Drug Administration (FDA) limit of 100 ppb. Both OTA and ZEA were found at low concentrations and were absent in some samples. This study suggested that higher priced dog food does not ensure superior quality or that it is free from contamination with fungi or mycotoxins. However, analysis of the more expensive PBs did reveal contamination concentrations lower than those of the SBs.

## Introduction

A global trend of increasing pet ownership, with the concurrent affluence of modern society, has led to a greater demand for specialised pet products and diets. Feeding of pets is influenced by societal habits and income. This market trend resulted in the development of many new products. The ingredients and nutritional content of most pet foods are adequately indicated on the label, and many pet food brands are registered and accredited with some consumer interest group, such as the Pet Food Institute (PFI), Association of American Feed Control Officials (AAFCO) or a governmental organisation (*South African Government: The Fertilizer, Farm Feeds, Agricultural Remedies and Stock Remedies Act* [No. 36 of 1947]). Dry pelleted pet food often contains 5% – 28% of animal protein or its derivatives with the remaining portion consisting of maize, maize gluten, wheat, wheat gluten, rice and its by-products amongst other ‘millings’ (Brown 1997; Klich & Pitt 1988; Krishnamachari et al. 1975). The remaining portion consists of vegetable matter, bulking agents and chemical additives (Brown 1997; Klich & Pitt 1988). However, none of these reflects the digestibility of the diets or content of chemical agents and potential toxins. In a highly competitive and price-driven pet food market, the use of inferior maize, maize gluten, wheat, wheat gluten, rice and its by-products is common. In addition, inferior-quality slaughterhouse renderings and milling by-products make up the fillers in the formulation of price-driven pet diets (Klich & Pitt 1988).

Examination of dog food packaging and product labels indicates that the claimed high crude protein content is largely vegetable in nature and minimally from meat sources. Packaging labels provide extensive information regarding ingredients, but with limited information on actual percentages of ingredients used in the formulation. Market segmentation often leads to misunderstanding amongst consumers about the nutritional value of the product. Cereal products that are considered unfit for human consumption are often incorporated into feed formulations and act as excellent substrates for the growth of microorganisms such as fungi. These contaminated cereals (Bennett & Klich 2003; Tulpule 1981) often become a health risk to pets, resulting in outbreaks of mycotoxicosis associated with morbidity and mortality. The cheaper feeds and ‘home industry’ preparations have often been implicated in acute and chronic mycotoxin poisoning (Arnot et al. 2012; Newman et al. 2007; Stenske et al. 2006) that resulted in severe clinical signs (which included depression, anorexia and weakness) and sudden death. Chronic mycotoxin exposure resulted in depression, anorexia, weakness and weight loss (Dereszynski et al. 2008; Newman et al. 2007).

We analysed dry pelleted dog food (both premium brands [PB] sourced from veterinary outlets sold at relatively higher prices [R40.00/kg – R100.00/kg] and foods purchased at non-veterinary outlets at relatively lower prices [SB] for R4.00/kg – R30.00/kg). The latter feeds are generally not endorsed by veterinarians. PB feeds are perceived to contain more protein from meat sources but, in reality, are not devoid of cereals and meat by-products, which also make them susceptible to mycotoxin contamination. SBs contain cereals and meat by-products as major ingredients. The general perception that the expensive PBs sold through veterinary clinics and specialised veterinary outlets are the best quality was challenged when compared to the SBs. Veterinary knowledge on the potential range of toxicological effects are still limited, although its serious health effects have been recorded and described (Boermans & Leung 2007; Bryden 2012). This study aimed to identify, quantify and compare fungal and some commonly found mycotoxin profiles for both marketing channels under recommended legislation.

## Materials and methods

### Materials

All chemicals, reagents and mycotoxin standards were obtained from Merck (South Africa) and Sigma (South Africa) unless otherwise specified. Twenty bags of dry pelleted dog food, range for adult dogs, were purchased from veterinary outlets (PB) (*n* = 10) and non-veterinary outlets (SB) (*n* = 10) in Durban, South Africa. Particular care was taken not to repeat brands while sampling. All bags were catalogued by brand name as well as serial and batch numbers. All samples were well within the expiry date listed on each pack. All mycotoxin standards were obtained from Sigma (St. Louis, USA). FB_1_ and FB_2_ were purchased from PROMEC (MRC, South Africa).

### Methodology

#### Sampling

All 20 bags (10 PB and 10 SB) of pelleted dog food were opened and mixed prior to obtaining a representative sample of 500 g. This representative sample was placed and sealed in a new clean plastic bag.

### Sample preparations

The sealed bags (500 g pelleted dog food) were well shaken, opened and a 200 g sample was weighed and placed in a blender jar. All feed samples were then milled to a fine powder using a mechanical blender (Petron 3600, Germany). The milled samples were used for fungal analysis and mycotoxin determination. Remaining samples were resealed and stored in individual sealed containers at 4 °C until required for further analysis.

### Fungal isolation

Fungal isolation was done by pipetting 1 mL of serially diluted 1 g blended material suspended in 9 mL Ringer’s solution on potato dextrose agar (PDA) and Ohio Agricultural Experimental Station agar (OAESA) (Kaufman, Williams & Sumner 1963) and sub-culturing of isolated colonies on PDA, malt extract agar (MEA) and Czapek yeast extract agar (CYA), followed by macro- and microscopic identification. Determination of each species of fungus was done using the keys of Klich and Pitt (1988) and Klich (2002) for *Aspergillus* spp. and Pitt and Hocking (1997) for *Penicillium* and other genera. This was done by observing both the macroscopic characteristics of the colonies on various media used and the microscopic morphology and measurements of the conidiophores (after staining mycelia with 0.1% fuchsin dissolved in lactic acid) under an Olympus B061 Compound microscope (Wirsam Scientific, South Africa) and Microscope Standard 19 (470919–9902/06), equipped with an Axiocam MRC Camera Ser. No. 2 08 06 0245 and AxioVision Release 4.5 SP1 (03/2006) software (Zeiss, West Germany).

### Mycotoxin extraction and clean-up of feed samples

A multi-mycotoxin extraction method (multi-mycotoxin screen) devised by Patterson and Roberts (1979) was used for the extraction of aflatoxins (B_1_ and B_2_), ochratoxin A (OTA) and zearalenone (ZEA). Twenty-five grams of milled dog food was extracted using aqueous acetonitrile containing potassium chloride and the toxins further extracted with dichloromethane with added sodium bicarbonate to obtain a neutral (N) fraction and after reacidification to obtain an acid (A) fraction. The N fraction was dialysed against 30% aqueous acetone overnight and then back-extracted into dichloromethane. The two fractions were evaporated and dried under a nitrogen gas stream and stored in sealed vials. Except for OTA that was in the A fraction, all other mycotoxins of interest were in the N fraction.

For fumonisins, the extraction and clean-up were done according to the method of Shephard and Sewram (2004) with minor modifications. A milled pelleted food sample (25 g) was extracted with 50 mL of methanol: water (3:1), and after shaking on a bench shaker (1 h), the entire content was filtered through a Whatman No. 2V filter paper. The filtrate was passed through a previously conditioned strong anion cartridge (SAX) column (Bond Elute, VARIAN, South Africa) with 5 mL methanol followed by 5 mL methanol: water (3:1 v/v). The column was washed with 8 mL methanol: water (3:1, v/v) and then 3 mL methanol. The absorbed fumonisins were then eluted with 10 mL 1% acetic acid in methanol. The eluent was evaporated and dried under nitrogen gas and the residue stored in a screw cap vial (4 °C) until analysed.

For confirmation, aflatoxins (AFB_1_, AFB_2_) were extracted using an immunoaffinity column (VICAM) using the VICAM method as follows: 5 g NaCl was added to samples of milled feed (25 g), mixed with 100 mL of methanol/water (80/20; v/v) and blended for 1 min. The extract was filtered successively through fluted filter paper, and 10 mL of the extract was diluted to 50 mL with water, mixed and filtered using a microfibre filter. Ten mL of the final filtered extract was passed through the immunoaffinity column followed by 10 × 2 mL of distilled water. Aflatoxins were eluted with 1 mL methanol, dried under nitrogen gas and stored until analysis.

### Thin-layer chromatography

To detect each mycotoxin of interest, two-dimensional thin-layer chromatography (TLC) was performed (Patterson & Roberts 1979). Briefly, into the vial containing mycotoxin extract, 20 µL of 200 µL DCM-containing extract solution was spotted on a 10 mm × 10 mm silica gel TLC plate and a two-dimensional TLC performed using appropriate mobile phases. After the mobile phase reached the top of the plate, the plate was air dried and viewed under UV light (for fluorescent detection spots) or treated with P-anisaldehyde and heated in the oven for a minute for fumonisin detection.

### High-performance liquid chromatographic analysis of feed sample extracts

Aflatoxins B_1_ and B_2_, ZEA, OTA and fumonisins (B_1_ and B_2_) were quantified in the appropriate fractions of the sample extracts by high-performance liquid chromatography (HPLC). The mycotoxin extracts were dissolved in 1 mL methanol and filtered through a 0.2-µm millipore filter; the final filtrate was used as the analyte. The chromatographic separation of analytes and standards was performed by passing through the symmetry column with an operational oven temperature of 30 °C.

AFB_1_ and AFB_2_ were individually determined using HPLC with fluorescence detection after post-column electrochemical derivatisation with bromine using a KOBRA cell (Chu 1991). The eluent was water/methanol (58:42 v/v) with the addition of 119 mg potassium bromide and 100 µL nitric acids (65%) per litre at an isocratic flow rate of 0.8 mL/min. The aflatoxins were detected using a scanning fluorescence detector at excitation and emission wavelengths of 360 nm and 440 nm, respectively. ZEA was analysed by fluorescent detection at excitation and emission wavelengths of 274 nm and 418 nm, respectively. The injection volume was set at 20 µL, whereas the mobile phase (acetonitrile/water [45:55 v/v]) was pumped at the rate of 1 mL/min. OTA analysis was measured using fluorescence detection (Allcroft et al. 1961). The mobile phase consisted of acetonitrile/water/acetic acid (50:48:2 v/v/v) that was pumped at a rate of 1 mL/min. Respective fluorescence excitation and emission wavelengths of 334 nm and 460 nm were used.

Fumonisin-containing extracts were reconstituted in methanol, and 50 µL aliquots derivatised with 250 µL of *o*-pthaldialdehyde (OPA), prior to separation on a reversed-phase HPLC system using fluorescence detection at excitation and emission wavelengths of 335 nm and 440 nm, respectively (Shephard & Sewram 2004). The isocratic mobile phase made up of 0.1 M dehydrated sodium dihydrogen orthophosphate/methanol (80:20) with pH adjusted to 3.5 using orthophosphoric acid was pumped at a rate of 1 mL/min. The injection volume was 50 µL.

For recovery, selected feed samples with known concentrations were spiked with 100 µg/kg of AFB_1_, AFB_2_, OTA and ZEA and 200 µg/kg of FB_1_ and FB_2_ for determination of recoveries. The mean recoveries obtained in triplicate were 98.2% and 96.5% for FB_1_and FB_2_, respectively; 95.5% and 89% for AFB_1_ and AFB_2_, respectively and 93.0% and 94.6% for ZEA and OTA, respectively (Table 1).

**TABLE 1:** High-performance liquid chromatography recovery of the selected mycotoxins after spiking with appropriate amounts of the pure standard.

Mycotoxin	Concentration spiked (µg/kg)	Concentration measured (µg/kg)	% Recovery
AFB_1_	100	95.5	95.5
AFB_2_	100	89.0	89.0
OTA	100	94.6	94.6
ZEA	100	93.0	93.0
FB_1_	200	196.4	98.2
FB_2_	200	193.0	96.5

All samples were analysed on Shimadzu Corporation (Kyoto, Japan) LC-20AB liquid chromatograph equipped with CBM-20A communication bus module, LC-20AB degasser, CTO-20A column oven, Nova-Pak 4 mm C18 reversed-phase analytical column (250 mm × 4.6 mm, 5 µm), SIL-20A auto sampler, RF-10AxL fluorescence detector, RID-10A refractive index detector and SPD-M20A photodiode array detector linked to LC solutions version 1.22 Software Release.

## Results

The results of mycotoxin assay and fungal culture of food samples are presented retrospectively in Tables 1 and 2.

**TABLE 2:** Fungal identification and their approximate quantitation (colony forming units) in premium brands and standard brands feeds after culture.

Fungal isolates (CFU/mL)	Fungal species	Premium	Standard
*Aspergillus*	*A. flavus*	***	**
	*A. fumigatus*	*	**
	*A. niger*	*	*
	*A. niveus*	*	-
	*A. ochraceus*	*	-
	*A. parasiticus*	**	**
	*A. penicilioides*	-	*
	*A. poae*	-	-
*Fusarium*	*F. graminearum*	**	***
	*F. verticilliodes*	*	*
*Penicillium*	*Penicillium* spp.	**	**
	*P. polonicum*	-	-
	*P. crustosum*	-	-
Other	*Rhizopus* spp.	+	-
	Unidentified microbe	-	-
	Yeast	+	+

* , 100 – 300 × 10^4^ CFU;

** , 300 – 500 × 10^4^ CFU;

*** , > 500 × 10^4^ CFU; **+**, Positive only.

The most prevalent fungal isolates in all dog samples were *Aspergillus* species, *Fusarium* species and *Penicillium* species (Table 2). The fungal isolates of *Aspergillus flavus, Aspergillus fumigatus* and *Aspergillus parasiticus* were the most prevalent in both categories but the PB showed highest levels of *A. flavus* (500 × 10^4^ CFU’s) as compared to the SB. The other fungal occurrences of *Aspergillus* species were similar for both categories. The SB had a higher number of fungal isolates for *Fusarium graminearium,* whereas *Fusarium verticilliodes* was similar for both categories. Isolates of *Penicillium* were similar for both PB and SB dog feeds. In general, there was a prevalence of a variety of fungal species in both feed categories with *Aspergillus* species that are commonly implicated in aflatoxicosis being the most abundant.

The rapid TLC method allows for quick identification of the mycotoxins present in the feed extracts (using Rf values and spiking with known standards). The PB and SB samples displayed the presence of all four mycotoxins evaluated. PBs showed higher concentrations of FB and lower concentrations for the other three mycotoxins, whereas SBs displayed moderate concentrations of AF and FB with low concentrations of OTA and ZEA.

All 20 samples were contaminated with AF. Both PBs (20.17 µg/kg) and SBs (44.17 µg/kg) showed high concentrations of AFs with AFB_1_ exceeding the concentration limit of 10 ppb limit regulated by the *Fertilizer, Farm Feeds, Agricultural Remedies and Stock remedies Act* (No. 36 of 1947) (South African Government 2009) and similar international standards (FDA 2001). Five PB and five SB samples exceeded the 10 ppb limit. Two SB samples exceeded 100 ppb for fumonisin, more than 10 times the permissible concentration. OTA and ZEA were detected in most samples at very low concentrations. ZEA is produced by *F. graminearium, F. nivale,* and *F. avenaceum* and has been implicated in the reproductive pathology of canines. OTA, a nephrotoxin, is produced by a number of *Aspergillus* and *Penicillium* species.

## Discussion

Companion animal ownership has evolved from being a peripheral addition to an integral part of modern human life. In the United States, the total cat and dog ownership is about 150 million and is worth $50 billion which translates to about 65% of households owning at least one pet, with pet ownership expected to grow annually by 4% – 5% (Pet Care Analysis 2017). The trend of humanisation has driven the pet food market forward as humans constantly demand quality foods and accessories for their companion animals. Companion animals have moved from being ‘outside pets’ to be members of the inner circle of human daily household life. The drive for convenient food products amongst consumers extends to their pets hence increasing animal welfare awareness. The consumer would rather buy conveniently packed pelleted or canned pet food than the raw ingredients (Pet Care Analysis 2017). The perception of higher priced pet foods found in the PB channel being of better quality was investigated. The analysis of dry pelleted food for major pathogenic fungi and mycotoxins in both marketing channels allowed us to compare and test this hypothesis.

Pet foods (cheaper and low quality sold via supermarkets and ‘home industry’) are known to contain more cereals and cereal by-products, leading to the common assumption that these would be heavily contaminated with fungi than the more expensive food brands. This ideology was found to be questionable based on the analysis of our 20 samples tested in both PB and SB marketing channels. This was of particular concern because both *A. flavus* and *A. parasiticus* were detected at high concentrations (between 300 and 500 × 10^4^ CFU’s). These fungi were previously implicated in AF outbreaks in domestic animals (Arnot et al. 2012; Fox, Hodgkins & Smart 2012). These fungi commonly produce AF that are potent hepatotoxins and hepatocarcinogens that may lead to serious clinical signs and even sudden death (Dereszynski et al. 2008; Fox et al. 2012; Newman et al. 2007). Dogs’ susceptibility to AFs is attributed to their low glutathione S transferase activity that plays an important role in detoxification of this mycotoxin (Dereszynski et al. 2008). In many AF outbreaks, dogs were found to be susceptible to low dose ranges of 50 µg/kg – 300 µg/kg for 42–48 days and high doses of 500 µg/kg – 1000 µg/kg body weight (BW) for acute cases (Lazicka & Orzechowski 2010). AFB_1_ is highly toxic compared to its other forms and has been the main aetiological cause of dog deaths at dose levels of 223 µg/kg – 579 µg/kg food resulting in severe liver failure (Krishnamachari et al. 1975; Newman et al. 2007). In our study, these levels often exceed tolerable levels for canines and have been implicated in many cases of aflatoxicosis (Arnot et al. 2012; Newman et al. 2007) (Table 1). Our findings are not surprising as these are ubiquitous soil fungi that contaminate agricultural crops like maize, groundnuts and other cereal grains (Leung, Diaz-Llano & Smith 2006) and are implicated in mycotoxicosis. The SB had a higher number of fungal isolates for *F. graminerium*, whereas *F. verticilliodes* was similar for both categories (Table 2). Fusarium mycotoxins are a broad and diverse group that have been implicated in a wide variety of clinical symptoms in animal toxicology (Placinta, D’Mello & Macdonald 1999). *Aspergillus* and *Penicillium* species are known to be implicated in ochratoxicosis, caused by the nephrotoxin OTA (Leung et al. 2006; Shephard & Sewram 2004). Recent investigations have lent support to a multi-aetiological syndrome with regard to mycotoxin poisonings. Publications emanating from South Africa (Arnot et al. 2012) and Israel (Fox et al. 2012) together with analytical work around pet foods and cereal ingredients (Fox et al. 2012; Mwanza et al. 2013) substantially support this idea.

Fumonisin and human disease seem to be poorly correlated, but feeds contaminated with *F. verticilliodes* (produce fumonisins especially FB_1_) have resulted in cardiotoxicity and cardiorespiratory signs in pigs (Harrison et al. 1990). The *Fusarium* sp. toxins, viz. fumonisin, ZEA and trichothecenes, are all implicated in adverse effects on animal health (Placinta et al. 1999). At this point, not much information is available on toxicity in dogs, and prescribed minimum concentrations in pet foods are unclear. However, in view of their contribution to serious disease in equines (leukoencephalomalacia), swine (hepatitis and pulmonary oedema) and rodents (hepatic and renal) (Placinta et al. 1999; Voss, Smith & Haschek 2007), their contribution to animal ill health and immunosuppression cannot be ignored (Boermans & Leung 2007; D’Mello, Placinta & Macdonald 1999). A single SB sample failed the 100 ppb limit set by the FDA for animal feeds. The FDA (2001) sets its limits between 100 ppb for equines and up to 5000 ppb for poultry, whereas Canada, Japan and many countries sets no limits for fumonisins in pet foods (FDA 2001). OTA is nephrotoxic in companion animals (Shephard & Sewram 2004). A study on beagle dogs demonstrated susceptibility and vulnerability to both acute (7.8 mg/kg BW) and chronic exposure at 200 µg/kg (Gourama & Bullerman 1995; Razzazi et al. 2001).

**FIGURE 1:**
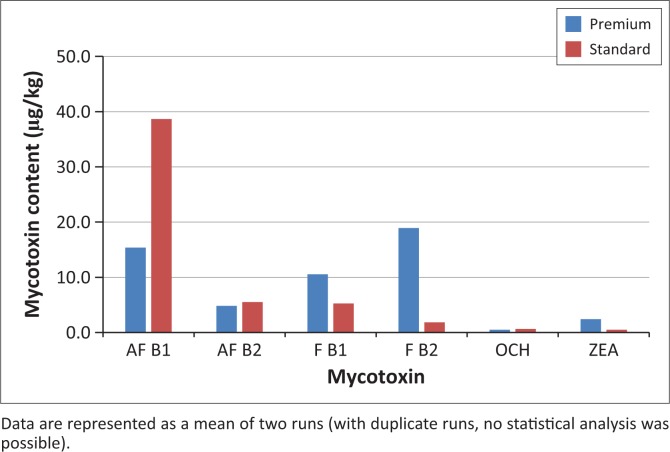
High-performance liquid chromatography quantitation of the four major mycotoxins investigated in this study.

OTA concentrations in both feed categories (PB and SB) were low (Table 1). Our study concurred with a similar study in Europe where OTA, although present in the mycotoxin mix, was found at much lower concentrations than AFs and FBs (Gajęcka et al. 2004). OTA, a nephrotoxin produced by a number of *Aspergillus* and *Penicillium* species, displayed a similar quantitation trend to that of ZEA (Leung et al. 2006; Shephard & Sewram 2004). Other studies found that OTA and ZEA were also less prevalent than AFs and FBs (Gajęcka et al. 2004; Lazicka & Orzechowski 2010) (Figure 1).

ZEA detection by HPLC was limited and when detected it was present in very low concentrations (2.4 µg/kg in PB and 0.5 µg/kg in SB) (Table 1). ZEA, a product of *F. graminearium, F. nivale* and *F. avenaceum*, has been implicated in reproductive pathology of canines (Rotimi et al. 2016). A study by Lazicka and Orzechowski (2010) found significant contamination with ZEA with a high concentration of 298 µg/kg, similar to other studies in Europe. ZEA toxicity correlated very closely with diseases of the reproductive system after exposure to 200 µg/kg BW of toxin for a week (Gajęcka et al. 2004).

Irrespective of the marketing channel or price range, all foods tested presented a potential health risk to dogs. This observation then begs the question: does price ensure a mycotoxin-free pet food? Do PBs ensure better and higher quality ingredients and quality control? Producers of superior brands of dog food claim that their ingredients used in the formulation of pet food are superior. The labelling requirement by both governmental and non-governmental groups require that the nutritional content and ingredients used in formulation be listed on the packaging. But this claim does not ensure mycotoxin-free ingredients, which remain an area of risk for the consumer and the pet (Fox et al. 2012). However, an important influencing factor for fungal growth and mycotoxin production is directly related to how the product is handled and stored post-harvest and manufactured in the case of pelleted dog food (Bryden 2012; Tulpule 1981). In South Africa, PB dog foods are imported from the United States or Europe and transported to SA by ship. It could be that this mode of transport may present an opportunity for the proliferation of fungi (the holding facilities may be damp and not well aerated) and their subsequent production of mycotoxins while in a container with high humidity and extremes of heat (Maia & Pereira Bastos de Siqueira 2002). The problem may be exacerbated by the high concentrations of nutrients in PB food that could provide an ideal substrate for the production of mycotoxins (Gourama & Bullerman 1995). This may explain the high levels of AFs in PB foods that exceeded the minimum limits prescribed (10 ppb). SB products are often price driven in a highly competitive market, and it is understandable that poor-quality ingredients are often used in its formulation. The significant levels of AFs and FBs in both PBs and SBs represent a potential risk in the mycotoxin mix present in dog foods globally.

## Conclusion

Our study shows that purchasing dog foods based on price and marketing channels does not ensure a mycotoxin-free product. It was surprising that PBs, in some cases, contained a higher mycotoxin content than SBs. Some SBs did, however have a few samples that exceeded the prescribed limit up to 10 times the limit set by the *Fertilizer, Farm Feeds, Agricultural Remedies and Stock Remedies Act* (No. 36 of 1947) (South African Government 2009). With a booming pet industry, opportunistic investors are always looking for quick return on investments. This subjects the pet food industry to economic predators that compromise quality for huge profit. This gives rise to the question of quality control in both procurement, feed formulation and production that requires further research and investigation. Concise labelling presents a challenge with present practice providing limited and often nebulous information. Label words such as ‘derivatives’ and ‘by-products’ are vague and do not reflect the true content of the feed formulation. With respect to mycotoxins, the ingredients should clearly state the quantity of cereals present with an indication of approximate levels of the most commonly occurring mycotoxins. Clear rules of engagement should be provided for storage of feeds (open and closed bags), and the implementation of HACCP principles in feed manufacture will improve the quality of the end product (Horchner & Pointon 2011). Further studies concerning the use of AF-free transgenic maize (Thakare et al. 2017) together with improved processing and packaging technology may provide a possible answer to our questions. These are serious ethical considerations surrounding animal welfare and food safety to companion animals.

## References

[CIT0001] Allcroft R., Carnaghan R., Sargeant K. & O’Kelly J, 1961, ‘A toxic factor in Brazilian groundnut meal’, *Veterinary Record* 73, 428–429.

[CIT0002] Arnot L.F., Duncan N.M., Coetzer H. & Botha C.J, 2012, ‘An outbreak of canine aflatoxicosis in Gauteng Province, South Africa’, *Journal of the South African Veterinary Association* 83, 1–4. 10.4102/jsava.v83i1.2 23327140

[CIT0003] Bennett J. & Klich M, 2003, ‘Mycotoxins’, *Clinical Microbiology Reviews* 16, 497–516. 10.1128/CMR.16.3.497-516.2003 12857779 PMC164220

[CIT0004] Boermans H.J. & Leung M.C, 2007, ‘Mycotoxins and the pet food industry: Toxicological evidence and risk assessment’, *International Journal of Food Microbiology* 119, 95–102. 10.1016/j.ijfoodmicro.2007.07.063 17889389

[CIT0005] Brown R.G, 1997, ‘A comparison of certified and noncertified pet foods’, *Canadian Veterinary Journal* 38(11), 707–712. 9360790 PMC1576833

[CIT0006] Bryden W.L, 2012, ‘Mycotoxin contamination of the feed supply chain: Implications for animal productivity and feed security’, *Animal Feed Science and Technology* 173, 134–158. 10.1016/j.anifeedsci.2011.12.014

[CIT0007] Chu F.S, 1991, ‘Mycotoxins: Food contamination, mechanism, carcinogenic potential and preventive measures’, *Mutation Research/Genetic Toxicology* 259, 291–306. 10.1016/0165-1218(91)90124-5 2017214

[CIT0008] Dereszynski D.M., Center S.A., Randolph J.F., Brooks M.B., Hadden A.G., Palyada K.S. et al., 2008, ‘Clinical and clinicopathologic features of dogs that consumed foodborne hepatotoxic aflatoxins: 72 cases (2005–2006)’, *Journal of the American Veterinary Medical Association* 232, 1329–1337. 10.2460/javma.232.9.1329 18447777

[CIT0009] D’Mello J., Placinta C. & Macdonald A, 1999, ‘Fusarium mycotoxins: A review of global implications for animal health, welfare and productivity’, *Animal Feed Science and Technology* 80, 183–205. 10.1016/S0377-8401(99)00059-0

[CIT0010] Food and Drug Administration (FDA), 2001, *US Department of Food and Drug Administration: Guidance for the industry: Fumonsin levels in human foods and animal feeds*, viewed 25 June 2017, form https://www.fda.gov/Food/GuidanceRegulation/GuidanceDocumentsRegulatoryInformation/ChemicalContaminantsMetalsNaturalToxinsPesticides/ucm109231.htm

[CIT0011] Fox M.W., Hodgkins E. & Smart M.E, 2012, *Not fit for a dog!: The truth about manufactured dog and cat food*, Linden Publishing, South Mary, Fresno, CA.

[CIT0012] Gajęcka M., Jakimiuk E., Polak M., Otrocka-Domagała I., Janowski T., Zwierzchowski W. et al., 2004, ‘Zearalenone applied per os provides adverse effects in structure of chosen parts of bitch reproductive system’, *Polish Journal of Veterinary Science* 7, 59–66. 15061487

[CIT0013] Gourama H. & Bullerman L.B, 1995, ‘*Aspergillus flavus* and *Aspergillus parasiticus*: Aflatoxigenic fungi of concern in foods and feeds: A review’, *Journal of Food Protection^®^* 58, 1395–1404. 10.4315/0362-028X-58.12.1395 31159052

[CIT0014] Harrison L.R., Colvin B.M., Greene J.T., Newman L.E. & Cole J.R, 1990, ‘Pulmonary edema and hydrothorax in swine produced by fumonisin B1, a toxic metabolite of *Fusarium moniliforme*’, *Journal of Veterinary Diagnostic Investigation* 2, 217–221. 10.1177/104063879000200312 2094448

[CIT0015] Horchner P.M. & Pointon A.M, 2011, ‘HACCP-based program for on-farm food safety for pig production in Australia’, *Food Control* 22, 1674–1688. 10.1016/j.foodcont.2011.03.028

[CIT0016] Kaufman D.D., Williams L.E. & Sumner C.B, 1963, ‘Effect of plating medium and incubation temperature on growth of fungi in soil-dilution plates’, *Canadian Journal of Microbiology* 9, 741–751. 10.1139/m63-100

[CIT0017] Klich M.A, 2002, *Identification of common Aspergillus species*, Centraalbureau voor schimmelcultures, Utrecht, The Netherlands.

[CIT0018] Klich M.A. & Pitt J, 1988, ‘Differentiation of Aspergillus flavus from A. parasiticus and other closely related species’, *Transactions of the British Mycological Society* 91(1), 99–108. 10.1016/S0007-1536(88)80010-X

[CIT0019] Krishnamachari K.A.V.R., Nagarajan V., Bhat R. & Tilak T.B.G, 1975, ‘Hepatitis due to aflatoxicosis: An outbreak in western India’, *The Lancet* 305(7915), 1061–1063. 10.1016/S0140-6736(75)91829-2 48730

[CIT0020] Lazicka K. & Orzechowski S, 2010, ‘The characteristics of the chosen mycotoxins and their toxic influence on the human and animal metabolism’, *Natural Science* 2(6), 544–550. 10.4236/ns.2010.26068

[CIT0021] Leung M.C., Diaz-Llano G. & Smith T.K, 2006, ‘Mycotoxins in pet food: A review on worldwide prevalence and preventive strategies’, *Journal of Agricultural and Food Chemistry* 54(26), 9623–9635. 10.1021/jf062363+ 17177480

[CIT0022] Maia P.P. & Pereira Bastos De Siqueira M, 2002, ‘Occurrence of aflatoxins B 1, B 2, G 1 and G 2 in some Brazilian pet foods’, *Food Additives & Contaminants* 19, 1180–1183. 10.1080/0265203021000011214 12623678

[CIT0023] Mwanza M., Ndou R.V., Dzoma B., Nyirenda M. & Bakunzi F, 2013, ‘Canine aflatoxicosis outbreak in South Africa (2011): A possible multi-mycotoxins aetiology’, *Journal of the South African Veterinary Association* 84, 1–5. 10.4102/jsava.v84i1.133 23905208

[CIT0024] Newman S.J., Smith J.R., Stenske K.A., Newman L.B., Dunlap J.R., Imerman P.M. et al., 2007, ‘Aflatoxicosis in nine dogs after exposure to contaminated commercial dog food’, *Journal of Veterinary Diagnostic Investigation* 19, 168–175. 10.1177/104063870701900205 17402611

[CIT0025] Patterson D. & Roberts B, 1979, ‘Mycotoxins in animal feedstuffs: Sensitive thin layer chromatographic detection of aflatoxin, ochratoxin A, sterigmatocystin, zearalenone, and T-2 toxin’, *Journal of the Association of Official Analytical Chemists* 62, 1265–1267. 521411

[CIT0026] Pet Care Analysis 2017 *Pet Care Industry Analysis 2017- Cost and Trends*, viewed 16 August 2017, from www.franchisehelp.com-reports/petcare-industry-report,2017

[CIT0027] Pitt J.I. & Hocking A.D, 1997, *Fungi and Food Spoilage*, 2nd edn., Blackie Academic & Professional, London.

[CIT0028] Placinta C., D’Mello J. & Macdonald A, 1999, ‘A review of worldwide contamination of cereal grains and animal feed with *Fusarium* mycotoxins’, *Animal Feed Science and Technology* 78, 21–37. 10.1016/S0377-8401(98)00278-8

[CIT0029] Razzazi E., Böhm J., Grajewski J., Szczepaniak K., Kübber-Heiss A. & Iben C, 2001, ‘Residues of ochratoxin A in pet foods, canine and feline kidneys’, *Journal of Animal Physiology and Animal Nutrition* 85, 212–216. 10.1046/j.1439-0396.2001.00331.x 11686791

[CIT0030] Rotimi O.A., Rotimi S.O., Oluwafemi F., Ademuyiwa O. & Balogun E.A, 2016, ‘Coexistence of aflatoxicosis with protein malnutrition worsens hepatic oxidative damage in rats’, *Journal of Biochemical and Molecular Toxicology* 30, 269–276. 10.1002/jbt.21787 26804159

[CIT0031] Shephard G. & Sewram V, 2004, ‘Determination of the mycotoxin fumonisin B1 in maize by reversed-phase thin-layer chromatography: A collaborative study’, *Food Additives and Contaminants* 21, 498–505. 10.1080/02652030410001670175 15204551

[CIT0032] South African Government, 2009, *Fertilizers, Farm Feeds, Agricultural Remedies and Stock Remedies Act (Act No.36 of 1947)*. South African Government Gazette No. R227, 2009 March 6, Government Printer, Pretoria.

[CIT0033] Stenske K.A., Smith J.R., Newman S.J., Newman L.B. & Kirk C.A, 2006, ‘Aflatoxicosis in dogs and dealing with suspected contaminated commercial foods’, *Journal of the American Veterinary Medical Association* 228, 1686–1691. 10.2460/javma.228.11.1686 16740069

[CIT0034] Thakare D., Zhang J.,Wig R.A., Cotty P.J. & Schmidt M.A, 2017, ‘Aflatoxin-free transgenic maize using host-induced gene silencing’, *Science Advances* 3, e1602382 10.1126/sciadv.1602382 28345051 PMC5345927

[CIT0035] Tulpule P, 1981, ‘Aflatoxins – Experimental studies’, *Journal of Cancer Research and Clinical Oncology* 99, 137–142. 10.1007/BF00412449 7251631 PMC12253725

[CIT0036] Voss K., Smith G. & Haschek W, 2007, ‘Fumonisins: Toxicokinetics, mechanism of action and toxicity’, *Animal Feed Science and Technology* 137, 299–325. 10.1016/j.anifeedsci.2007.06.007

